# Structure of the Ulster Strain Newcastle Disease Virus Hemagglutinin-Neuraminidase Reveals Auto-Inhibitory Interactions Associated with Low Virulence

**DOI:** 10.1371/journal.ppat.1002855

**Published:** 2012-08-09

**Authors:** Ping Yuan, Reay G. Paterson, George P. Leser, Robert A. Lamb, Theodore S. Jardetzky

**Affiliations:** 1 Department of Structural Biology, Stanford University School of Medicine, Stanford, California, United States of America; 2 Department of Molecular Biosciences, Northwestern University, Evanston, Illinois, United States of America; 3 Howard Hughes Medical Institute, Northwestern University, Evanston, Illinois, United States of America; Institut Pasteur, France

## Abstract

Paramyxovirus hemagglutinin-neuraminidase (HN) plays roles in viral entry and maturation, including binding to sialic acid receptors, activation of the F protein to drive membrane fusion, and enabling virion release during virus budding. HN can thereby directly influence virulence and in a subset of avirulent Newcastle disease virus (NDV) strains, such as NDV Ulster, HN must be proteolytically activated to remove a C-terminal extension not found in other NDV HN proteins. Ulster HN is 616 amino acids long and the 45 amino acid C-terminal extension present in its precursor (HN_0_) form has to be cleaved to render HN biologically active. Here we show that Ulster HN contains an inter-subunit disulfide bond within the C-terminal extension at residue 596, which regulates HN activities and neuraminidase (NA) domain dimerization. We determined the crystal structure of the dimerized NA domain containing the C-terminal extension, which extends along the outside of the sialidase β-propeller domain and inserts C-terminal residues into the NA domain active site. The C-terminal extension also engages a secondary sialic acid binding site present in NDV HN proteins, which is located at the NA domain dimer interface, that most likely blocks its attachment function. These results clarify how the Ulster HN C-terminal residues lead to an auto-inhibited state of HN, the requirement for proteolytic activation of HN_0_ and associated reduced virulence.

## Author Summary

Newcastle disease virus (NDV) can cause severe disease in birds, with the most virulent strains causing sudden death, even in vaccinated populations in the poultry industry. Highly virulent exotic NDV (END) strains have caused largescale outbreaks in the US in 1971 and 2003, requiring the culling of 12 million and 3 million chickens, respectively. Additional economic costs were associated with containment and cleanup. NDV strains vary greatly in their virulence and ability to cause such outbreaks. Two proteins at the surface of the virus, the hemagglutinin-neuraminidase (HN) and the fusion (F) protein, activate NDV entry into cells and variations in both proteins are linked to differences in strain-specific virulence. Certain avirulent strains of NDV, such as NDV Ulster, express a longer HN protein with a C-terminal segment that must be proteolytically cleaved to fully activate the protein. Here we demonstrate that the extra C-terminal 45 amino acids of NDV Ulster HN adopt a well-defined structure, not present in the shorter HN proteins from virulent strains, that blocks two key receptor binding regions necessary for attachment to cells and virus entry. The results clarify how this unique evolutionary adaptation suppresses HN functions in avirulent NDV strains, consistent with an important role for this region in modulating NDV pathogenicity.

## Introduction

Newcastle disease virus (NDV) belongs to the large and diverse family of paramyxoviruses, which is responsible for many human and animal diseases [1]. The paramyxoviruses include other viruses such as mumps virus, measles virus, Sendai virus, respiratory syncytial virus (RSV), metapneumovirus (MPV), parainfluenza viruses (PIV) 1–5, Nipah virus and Hendra virus. NDV infects birds and many different strains have been isolated worldwide that vary in pathogenicity and virulence. Highly virulent strains can cause a contagious disease with respiratory, neurological and digestive tract pathology, with the most severe infections leading to substantial economic losses in the poultry industry, despite aggressive vaccination programs. Highly virulent velogenic viscerotropic NDV strains, known as exotic NDV (END) strains, not endemic in the US, have caused severe disease outbreaks, such as the 1971 outbreak that required the killing of over 12 million chickens at a cost of $56 million with additional costs over a 4 year cleanup. A more recent END outbreak in 2002–2003 required the culling of over 3 million chickens in California at a cost of over $161 million. Continuing concerns about the severe economic impact of NDV outbreaks has led to the classification of NDV strains with an intracerebral pathogenicity index of >0.7, or containing a fusion protein with a multi-basic cleavage site, as a U.S. Department of Agriculture Select Agent [2], [3]. Recently it has been shown that NDV is able to selectively kill tumor cells, suggesting it could be useful as an oncolytic agent, and NDV is also being investigated as a potential vaccine vector (reviewed in [4]).

Paramyxoviruses are enveloped, negative-sense, single-stranded RNA viruses. Virions consist of a nucleocapsid, a matrix protein, and an envelope formed by a lipid membrane, typically with two glycoproteins displayed on the surface [1]. For virus penetration into target cells, the lipid envelope must fuse with a cell membrane. Membrane fusion, for nearly all paramyxoviruses, is triggered at the cell surface in a receptor-dependent, pH-independent manner, unlike the pH-dependent influenza virus hemagglutinin mechanism [1], [5], [6]. For most members of the virus family, two viral glycoproteins are required to mediate this entry process – the fusion (F) glycoprotein and an attachment protein referred to as hemagglutinin-neuraminidase (HN), hemagglutinin (H), or glycoprotein (G), depending on the virus [1], [5], [6]. Activation of the F protein requires virus-specific (homotypic) interactions with the attachment glycoprotein for viral entry, except for RSV and MPV [7], [8].

The HN attachment proteins are found in a subset of the paramyxoviruses, including NDV, mumps virus, parainfluenza virus 5 (PIV5), Sendai virus, and human parainfluenza viruses 1–4 (hPIV1–4) [1]. HN protein binds to the receptor (sialic acid) for virus attachment to cells and plays additional roles in the virus life cycle including F activation, and receptor-destroying (neuraminidase) activity to facilitate virus budding. HNs are type II membrane proteins, with N-terminal transmembrane domains followed by a stalk region and a large C-terminal globular head domain. The active form is thought to be a tetramer [1]. The four-helix bundle (4HB) stalk structures of the NDV Australia-Victoria (AV) HN [9] and the PIV5 HN [10] have been determined and revealed a surface site in the HN stalk that is thought to interact directly with F. Mutations at this HN stalk site affect both F protein binding and fusion activation (reviewed in [11]).

The severity of NDV-associated disease depends on the virus strain and the host species. NDV strains are grouped into three main pathotypes of high (velogenic), intermediate (mesogenic), or low (lentogenic) virulence. Both F and HN proteins are important determinants of the virus virulence [12], [13], [14], [15], [16], [17], [18]. In general, the HN proteins from different paramyxoviruses exhibit high sequence similarity, particularly in the core neuraminidase (NA) domain. The sequence of HN of NDV is overall highly conserved among isolates but one striking example of a difference occurs in lentogenic (low virulence) strains, such as the Ulster, D26 and Queensland strains that are 616 amino acids in length [19], [20], [21]. This is longer than most other NDV HN proteins (571, 577 amino acids), due to a C-terminal extension of 45 amino acids. Proteolytic cleavage of the C-terminal extension in HN_0_ removes 42 residues [19], leading to increases in neuraminidase and hemadsorption activities that are necessary for the viral life cycle [14], [15].

To understand better how the Ulster HN C-terminal extension down-regulates HN activities and may reduce virus pathogenicity, we determined the crystal structure of the NDV Ulster HN “head” or “NA” domain precursor. The structure reveals that the 45 amino acid extension crosses the outside of the NA domain and inserts C-terminal residues into the active site, interacting with residues involved in sialic acid receptor binding. The C-terminal extension also occludes a secondary sialic acid binding site implicated in virus attachment, effectively blocking both catalytic and binding functions of NDV HN. The C-termini are stabilized by an interchain disulfide bond formed by C596 and mutagenesis of this residue releases the inhibition of neuraminidase and hemadsorption activities. The results clarify the auto-inhibitory functions of the C-terminal extension present in Ulster HN and many other low virulence NDV strains.

## Results

### NDV Ulster HN_0_ cleavage is required for receptor binding and hemadsorption

**Figure 1:**
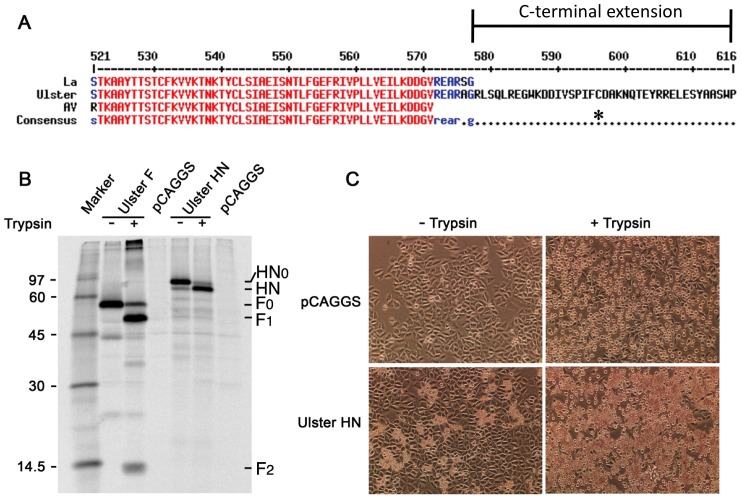
Recombinant NDV Ulster F and HN are expressed as F_0_ and HN_0_ precursors. (A) Sequence alignment of C-termini of NDV HN strains, La Sota (La), Ulster and Australia-Victoria (AV). Core NA domain residues are colored red, partially conserved C-terminal residues are colored blue. The cysteine at residue 596 is indicated by an asterisk. (B) NDV Ulster F and HN expressed from cDNAs in HeLa cells were metabolically labeled, immunoprecipitated and polypeptides analyzed by SDS-PAGE. Addition of trypsin to the transfected cells led to processing of the precursor proteins (F_0_ and HN_0_) as shown by the cleavage of F_0_ to F_1_ and F_2_ and the faster mobility of HN as compared to HN_0_. (C) Hemadsorption assay on HeLa cells expressing Ulster HN. Cells were overlaid with chicken red blood cells and subsequently washed with PBS^+^ and incubated at. 4°C. pCAGGS is vector control. Ulster HN expressed in HeLa cells binds sialic acid on chicken red blood cells very poorly. Treatment of HN_0_ with trypsin causes a large increase in red blood cell binding.

In the NDV Ulster strain, both HN and F proteins are initially produced as precusor proteins, F_0_ and HN_0_, which must be proteolytically cleaved to be fully active and support viral replication [14], [15]. The Ulster HN C-terminal extension lengthens the protein to 616 residues and contains many charged as well as aromatic amino acids (Figure 1A). To confirm prior observations obtained with virally expressed proteins, recombinant Ulster F and HN were expressed from cDNA by transfection of HeLa-CD4-LTR-β-gal (HeLa) cells with plasmids expressing the F and HN genes. The F and HN proteins were immunoprecipitated from [^35^S]-labeled lysates using rabbit polyclonal antibodies specific for F or HN and the precipitated proteins were analyzed by SDS-PAGE. The recombinant F and HN proteins were not cleaved efficiently but were detected as the precursors F_0_ and HN_0_ (Figure 1B). Both precursors could be cleaved extracellularly by the addition of exogenous trypsin, to produce F1, F2 and HN, as shown previously for the F and HN proteins synthesized in virus-infected cells [14], [15].

The receptor binding activity of the recombinant HN protein was examined using a hemadsorption assay as described in Materials and Methods. Without trypsin treatment, HeLa cells expressing Ulster HN protein exhibited a low level of chicken red blood cell (RBC) binding (Figure 1C, bottom left). Trypsin treatment of the HN expressing HeLa cells, prior to the addition of RBCs, greatly increased RBC binding (Figure 1C, bottom right). The results of a quantitative hemadsorption assay are shown in Table 1, further confirming these observations. The low level of binding activity detected in the absence of trypsin treatment is presumed to be due to the small amount of HN that is cleaved intracellularly as observed previously for virus-infected cells [14], [15].

**Table 1:** Hemadsorption of WT and C596S HN proteins.

	Hemadsorption (A 540 nM)
Ulster HN WT	0.122
Ulster HN WT+trypsin	0.329
Ulster HN C596S	0.466
Ulster HN C596S+trypsin	0.333

Hemadsorption was assayed by measuring the hemoglobin absorbance at 540 nM.

The values shown are the average of three experiments performed in duplicate.

### The NDV Ulster HN forms an additional interchain disulfide bond through C596 in the C-terminal extension

**Figure 2:**
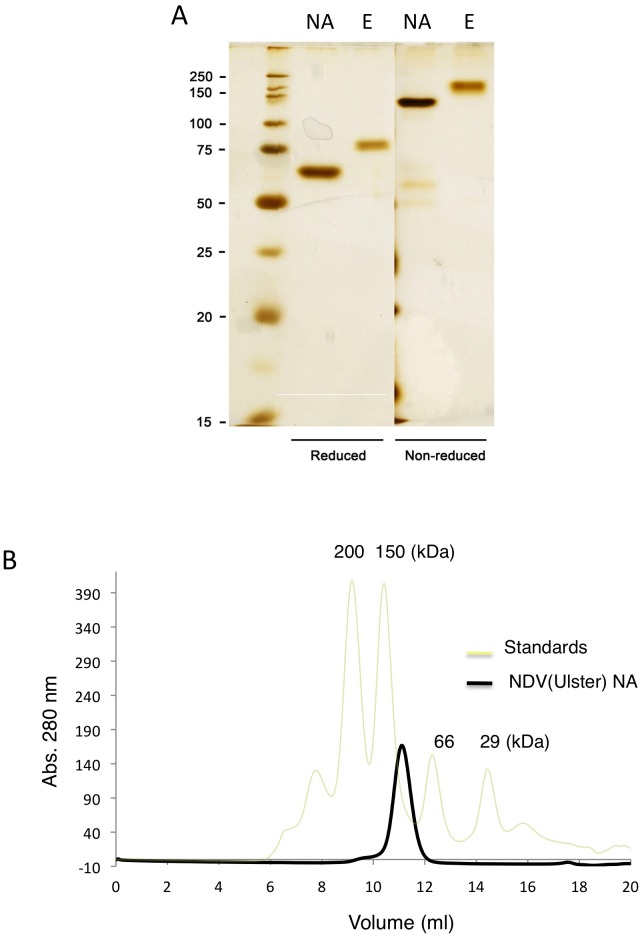
The NDV Ulster HN ectodomain and NA domain form disulfide linked dimers. (A) The NDV HN Ulster NA domain and the Ulster HN ectodomain were expressed in 293T cells and purified from the supernatant medium. Both HN NA domain (NA) and ectodomain (E) form dimers, as observed on silver stained SDS-PAGE gels under non-reducing conditions. Addition of reducing agent reduces the disulfide bond to yield monomers on SDS-PAGE. (B) Purified Ulster HN NA domain protein migrates with an apparent dimer molecular weight in gel filtration chromatography.

The ectodomain (residues 49–616) and NA domain (residues 124–616) of Ulster HN were expressed as soluble, secreted proteins in monolayers of 293T cells and purified as described in Materials and Methods. The proteins were >90% pure after elution from a Ni-NTA agarose column (Figure 2A). The construct of the NDV Ulster HN NA domain begins at residue 124, 1 residue after a conserved cysteine (C123) that forms an interchain disulfide bond. Previous studies have demonstrated that secreted paramyxovirus HN proteins form dimers and tetramers, but that isolated NA domains are primarily monomeric in solution [22], [23], [24], [25], [26], [27]. However, both the full length Ulster HN ectodomain, containing C123, and the shorter NA domain, lacking C123, form disulfide-linked dimers. Both the ectodomain and NA domain proteins contain disulfide bonds that can be reduced by dithiothreitol treatment (Figure 2A). The purified NA domain protein eluted with an apparent molecular weight of 107 kDa in gel filtration chromatography (Figure 2B), consistent with covalent dimer formation.

**Figure 3:**
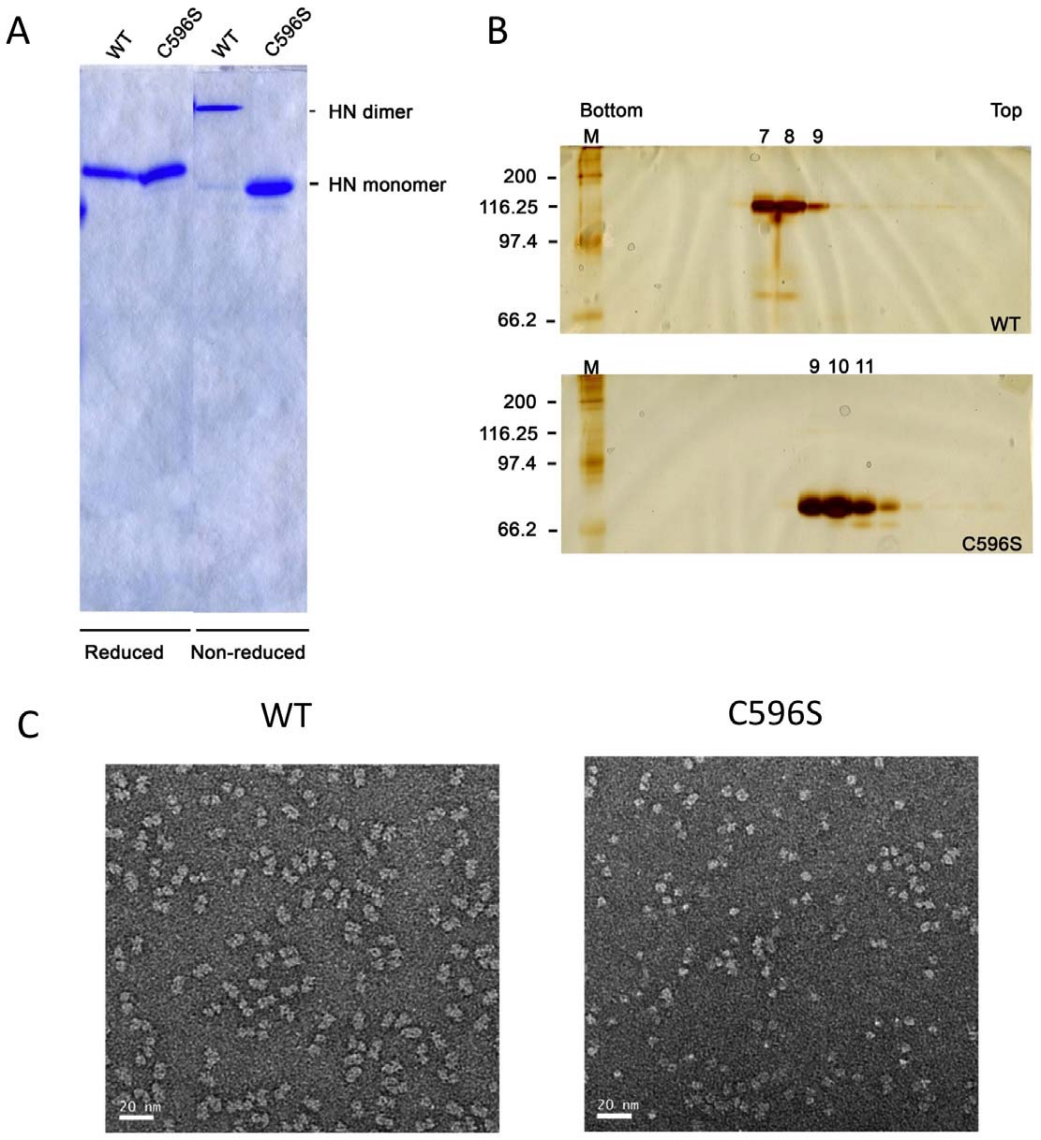
C596 in the HN C-terminal extension mediates NA domain dimerization. (A) HN Ulster WT NA domain and HN C596S NA domain were expressed and purified as described in Methods and analyzed by SDS-PAGE under reducing and non-reducing conditions. The gel was stained with Coomassie Brilliant Blue. (B) Sucrose density gradient sedimentation and SDS-PAGE (non-reducing conditions) of Ulster HN NA domain and HN C596S NA domain proteins. (C) EM of WT Ulster HN NA domain, indicates the protein consists primarily of dimers, and the HN C596S NA domain, consists mostly as monomers.

The C-terminal extension contains an additional cysteine (C596) not present in HN proteins containing 571 or 577 amino acids. The additional cysteine residue could account for the NA domain dimer formation (Figure 1A). To investigate the role ofC596 in forming a disulfide bond, we generated a mutant in which C596 was replaced by serine (C596S). C596S migrates as a monomer when analyzed by SDS PAGE under non-reducing conditions (Figure 3A). Further evidence that C596 forms an interchain disulfide bond was provided by sucrose gradient sedimentation and also electron microscopy of WT HN and C596S NA domains. Fractionation of sucrose gradients showed that the WT HN protein was found mainly in fractions 7–8, whereas C596S was mostly detected in fractions 9–11 (Figure 3B), consistent with their migration as dimers and monomers, respectively, on SDS-PAGE. Comparison of the sedimentation of HN and HN C596S with tetrameric, dimeric and monomeric forms of a soluble version of influenza virus neuraminidase (NAF) [28] further indicated that the WT HN NA domain sedimented as a dimer and the HN C596S NA domain sedimented as a monomer. In electron micrographs of negatively stained protein, the WT NA domain is observed mostly as dimers (Figure 3C, left) whereas the HN C596S mutant is observed mostly as monomers (Figure 3C, right), in agreement with the biochemical data.

We tested the neuraminidase activity of WT HN and HN C596S NA domains as described in Materials and Methods. Mutation of C596S results in a >3-fold increase in neuraminidase activity (Table 2). The C596S mutant was incorporated into the full length Ulster HN for receptor binding studies. As shown in Table 1, the mutation of C596S increased hemadsorption activity to levels comparable to trypsinized WT HN, with C596S binding activity being independent of trypsin. The interchain disulfide bond formed by C596 is therefore critical for maintaining the auto-inhibited state of the Ulster HN.

**Table 2:** Neuraminidase activity of the Ulster WT and C596S proteins.

	NA Activity (Em 440 nm)
HN WT Head	17369
HN C596S Head	54802

The neuraminidase activity of the Ulster WT and C596S proteins was assayed by measuring the fluorescence, at an emission wavelength of 440 nm, produced upon cleavage of the fluorogenic substrate 4-methylumbelliferyl-N-acetyl-α-D-neuraminic acid. The fluorescence shown was produced by equivalent amounts of protein.

### The extended C-terminus of the NDV Ulster HN is observed in a neuraminidase tetramer structure

**Figure 4:**
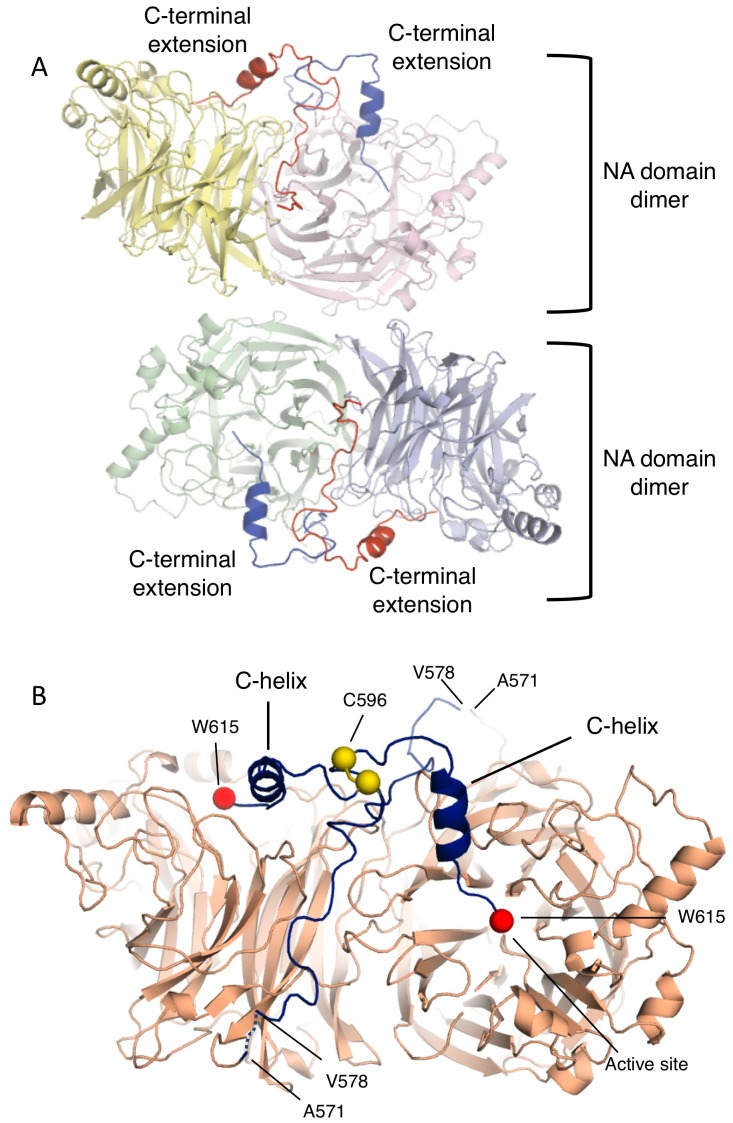
The inhibitory C-terminal extension forms a well-defined structure in crystals of the Ulster NA domain. (A) Crystal structure of the Ulster HN NA domain dimer, shows a dimer-of-dimers tetramer. The C-terminal extension is highlighted in dark blue and red in each pair of dimers. (B) The Ulster HN dimer is shown, with the C-terminal extension in dark blue. The C-terminal extension begins at the base of the β-propeller domain, extends along the outside of the dimer interface, and then rises above the active site before inserting the C-terminus into the receptor-binding site.

**Figure 5:**
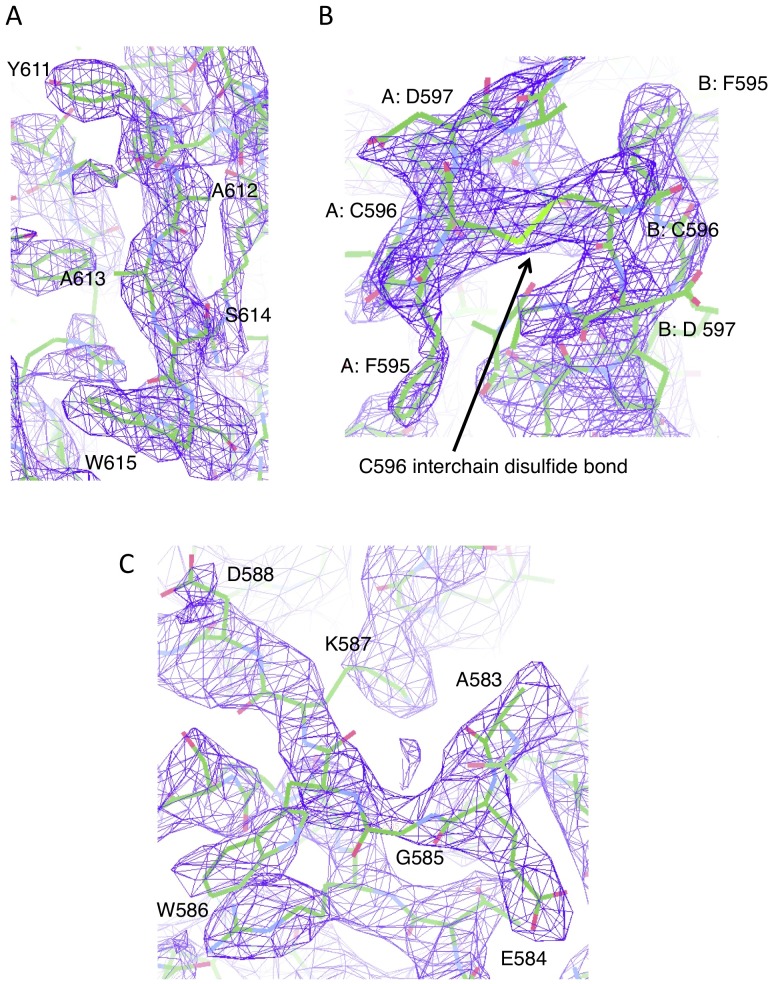
Electron density maps for key regions of the C-terminal extension. (A) Omit map electron density for C-terminal residues 611–615, which insert into the NA domain active site. (B) Omit map electron density for residues surrounding the interchain disulfide bond at C596. Residues associated with different chains of the observed NA domain dimers are indicated with an A: or B: prefix. (C) Omit map electron density C-terminal extension residues 583–588, which engage a second sialic acid binding site. The omit maps was calculated using the SFCHECK [46] program and contoured at 1.57 sigma.

The purified NA domain protein crystallized in space group P3121 and diffracted X-rays to 3.5 Å resolution (Table 3). The structure was solved by molecular replacement, using models of the NDV Kansas NA domain [25], [26], [29]. Four copies of the NA domains are located in the crystallographic asymmetric unit, arranged as an apparent dimer-of-dimers, reminiscent of other HN structures (Figures 4A, 5). The NA domain dimers are highly similar to those observed in structures of NDV Kansas HN [26], [29], hPIV3 HN [30], and PIV5 HN [22]. The apparent tetramer arrangement of the dimer-of-dimers differs from the NDV AV HN structure [9], which also contains its stalk region, but is similar to that observed for PIV5 HN [22]. Although two dimers pack into this tetramer arrangement, the Ulster HN ectodomain, like AV HN [9], forms dimers in solution, in contrast to the stable tetramers observed for PIV5 HN [22], [23].

**Table 3:** Crystallographic data and refinement statistics.

**Data collection**	
Source	APS NE-CAT 24-IDE
Wavelength (Å)	0.97916
Spacegroup	P31 2 1
Unit-cell parameters	
a (Å)	124.6
b (Å)	124.6
c (Å)	284.9
α (°)	90
β (°)	90
γ (°)	120
Completeness (%), overall/3.5 Å shell	99.3/100.0
Redundancy, overall/3.5 Å shell	3.6/3.7
Rfactor, overall/3.5 Å shell	0.115/0.69
I/sigma I, overall/3.5 Å shell	9.1/1.3
**Refinement**	
Resolution (Å)	47-3.5
No. reflections used (work/free)	32818/1662
R_work_/R_free_ (%)	0.237/0.304
No. of residues/atoms	1969/15242
Average B-factors (Å^2^), overall	162.0
Average B-factors (Å^2^), chains A/B/C/D/	126.5/139.4/167.9/214.3
R.m.s. deviations in bond lengths (Å)	0.006
R.m.s. deviations in bond angles (°)	1.04
Ramachandran plot statistics	
Residues in preferred regions (%)	92.2
Residues in allowed regions (%)	7.7

Electron density maps clearly show the presence of a majority of the C-terminus with a short main chain break at residue 571 (Figure 4A, B). The C-terminal extension wraps around the external surfaces of the HN dimer-of-dimers. The cleavage site for the activation of the precursor HN_0_ is located within the break in the electron density at residues 571–578 (Figures 1A, 4B), consistent with this region being flexible and accessible to proteolytic digestion [12], [14], [15], [19]. The extra C-terminal residues adopt a mostly extended conformation beginning near the bottom of the β-propeller structure, traversing the NA dimer interface, rising above the NA domain and inserting C-terminal residues in the HN catalytic site (Figures 4, 5A). A short helix (C-helix) is formed by residues 604–611 (Figure 4B). The four C-terminal extensions adopt very similar conformations, with RMSD values after superposition ranging from 0.27–0.65 Å. Two C-terminal extensions from adjacent subunits meet at C596, approaching from opposite directions. Electron density for the C596 interchain disulfide bond was observed, consistent with the biochemical and mutational data indicating the presence of the disulfide linkage.

### The Ulster HN C-terminus blocks the NA domain active site

**Figure 6:**
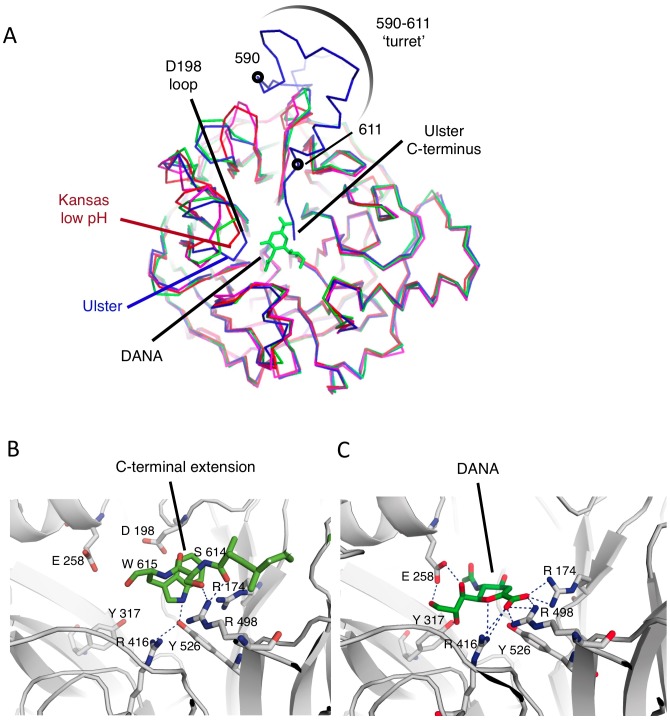
The C-terminal extension inhibits the NA domain active site. (A) Top view of a single HN NA domain subunit, showing superpositions with the NDV Kansas low-pH and DANA-bound crystal structures. The additional residues of the C-terminal extension are highlighted in blue and overlap the ligand bound in the active site. The D198 loop is indicated, which changes conformation in the Ulster HN structure, contributing to blocking the active site. (B,C) Detailed comparison of contacts made by the Ulster HN C-terminus and DANA sialic acid within the active site. Dotted lines represent hydrogen bonds and polar contacts identified by Pymol.

The Ulster HN C-terminal extension inactivates the NA domain catalytic site through three potential mechanisms (Figures 5A, 6A). First, the extreme C-terminal residues occupy the active site, forming specific interactions with residues involved in sialic acid binding. Second, an active site variable loop (the D198 loop) shifts into an inactive conformation, further inhibiting NA binding and catalytic activities. Third, residues in the C-terminal extension positioned outside and above the active site (the 590–611 ‘turret’) provide additional steric hindrance to the binding of larger oligosaccharide structures.

The interactions of the C-terminal residues that block the NA active site bury ∼350 Å^2^ of surface area and involve both polar and non-polar contacts (Figure 6B). The side chain of W615 is buried deepest within the active site, forming a hydrogen bond with Y526 and hydrophobic contacts with multiple residues, including R498, R416, D198, I175, and R174. S614 forms a hydrophobic contact with R498, while the main chain of A613 forms a hydrogen bond with R498. A612, which is positioned at the edge of the active site, forms a hydrophobic contact with A497. Y611, at the end of the C-helix, anchors the entry of the remaining residues into the active site. Many of these active site residues, R174, R498, R416, and E258, are directly involved in the interaction with sialic acid or a sialic acid inhibitor (2-deoxy-2,3-didehydro-N-acetylneuraminic acid or DANA), as revealed in the structures of NDV Kansas [29] (Figure 6C) and PIV5 HN [22]. Therefore, the insertion of the C-terminal residues into the active site directly disrupts receptor binding and NA activity. Cleavage of HN_0_ removes an 8kD glycopeptide corresponding to the C-terminal extension, which is not found in the mature protein [12], [14], [15], [19]. We have shown that mutation of the C596 disulfide bond also releases the auto-inhibited state. Both observations indicate that the interactions of residues 612–615 on their own are likely weak and insufficient to mediate inhibition in the absence of the remainder of the supporting C-terminal extension structure.

Although the NDV Ulster HN structure is overall very similar to the NDV AV [9] and Kansas HN structures [26], [29], one variable active site loop, the D198 loop comprising R197-S200, shows significant structural differences (Figure 6A). D198, which is equivalent to D151 in influenza virus NA, is thought to be directly involved in the hydrolysis of the glycosidic bond via a water molecule. In the Ulster HN structure, the D198 loop switches conformation, pointing deeper into the active site, and forms a hydrophobic contact with W615 (Figure 6B). In this conformation, the side chain of D198 would clash with the sialic acid substrate. In a low pH, ligand-free structure of NDV Kansas HN, the D198 loop also points into the active site [26], but not as deeply as observed in the Ulster HN structure (Figure 6A). The Ulster HN crystals were grown at pH 7.5, therefore the conformation of the D198 loop is not linked to an acidic pH condition and is more likely a result of the insertion of the C-terminus, and W615, into the active site. Together with the C-terminal residues (A612-P616: electron density for P616 was weak), the conformational change in the D198 loop would occupy the active site, fully blocking receptor engagement and catalytic activity.

The I590-Y611 region of the C-terminal extension forms a projection, or ‘turret’, to one side of the NA domain and above the active site (Figure 6A). Residues 590–611 could provide additional steric interference that would limit the ability of HN to bind larger, branched oligosaccharide structures present at the cell surface.

### An intersubunit hydrophobic core surrounding C596 props up the C-terminal extension and stabilizes its entry into the active site

**Figure 7:**
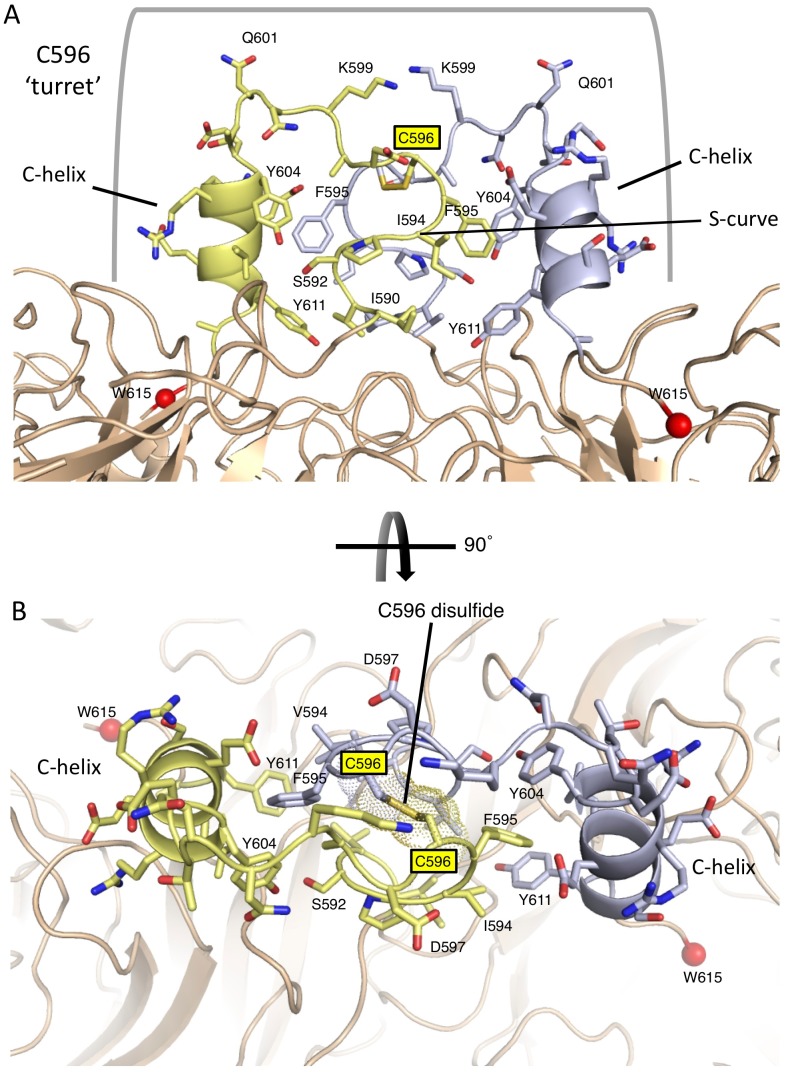
The C596 disulfide bond is located at the center of a turret-like projection formed by the C-terminal extension. (A) Residues 590–611 of the C-terminal extension form extensive intersubunit interactions centered around C596. Residues are colored yellow or blue from the respective subunits and key residues are labeled. The observable C-terminal residue W615 is indicated with a red Cα sphere and marks the position of the two active sites: there was no interpretable electron density for the C-terminal P616 residue. (B) View of the C596 disulfide bond at the crossover between subunits of the NA domain dimer. The C596 disulfide bond atoms are further indicated with dot surface representations.

The disulfide bond formed by C596 is critical for the Ulster HN auto-inhibition and is located at the center of a ‘turret’ supported by an extensive hydrophobic interface between C-terminal extensions of adjacent HN subunits (Figures 5B, 7A). The C-terminal extension forms symmetric intersubunit interactions through an S-curve structure as it rises above the β-propeller domain through residues 590–599. Residue 596 defines the center of the top of this turret structure, and the polypeptide chain then descends into the NA domain active site, forming the C-helix through residues 604–611. Residues 590–611 from the adjacent subunits form a set of interactions that support the turret and C-terminal helix, guiding the polypeptide chain into the active site (Figure 7A). Residues 590–595 from one subunit insert underneath the α-helix from the second subunit, interacting with its hydrophobic face.

Aromatic residues F595, Y604 and Y611 are central to this inter-subunit hydrophobic core (Figure 7A,B). I594 and F595 from one subunit pack against Y604 and Y611 from the second subunit. Additional hydrophobic core residues include I590 and L608, while S592 appears to form a hydrogen bond with Y604. Y611 packs at the lip of the active site, and is likely critical for stabilizing the position of the C-terminus and inhibition of receptor binding (Figure 7A,B). The C596 disulfide bond is centrally located in the crossover junction between the subunits, consistent with its critical role in auto-inhibition (Figure 7B). Mutation of C596 to serine likely destabilizes this structure, leading to displacement of the C-terminus from the active site and restoration of neuraminidase and hemadsorption activities.

### The C-terminal extension occludes a second sialic acid binding site

**Figure 8:**
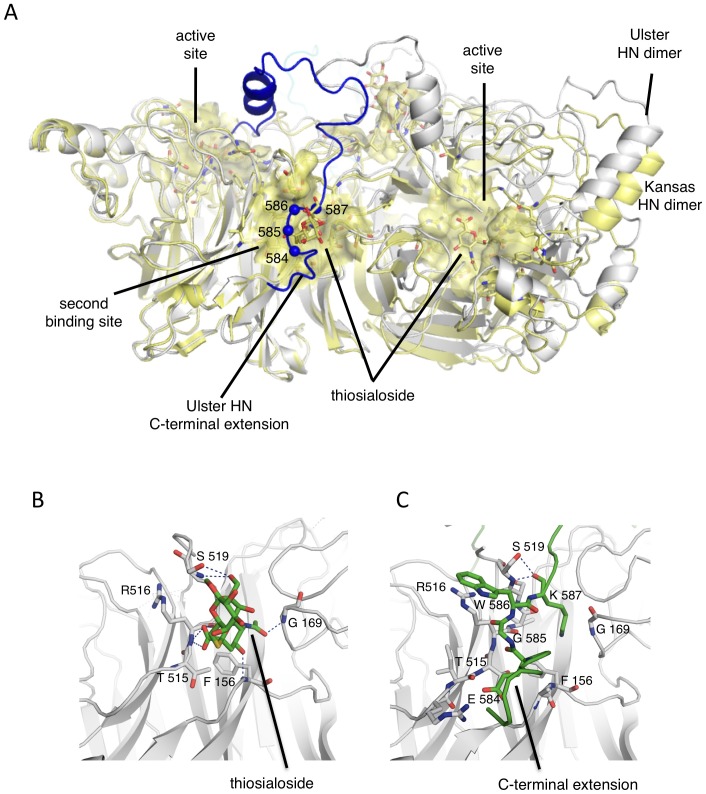
Residues 584–587 of the C-terminal extension engage the second sialic acid binding site at the NA domain dimer interface. (A) Superposition of Ulster (grey) and Kansas (yellow) NA domain dimers, highlighting the location of the second sialic acid binding site at the dimer interface. The Kansas and Ulster dimers superimpose with a RMSD of 1.6 Å. The active sites and second binding site are indicated with transparent yellow surfaces and labeled. Residues 584–587 of the C-terminal extension cover the second receptor binding site and are highlighted with blue Cα spheres. Comparisons of the detailed interactions of thiosialoside (B) and the C-terminal extension residues (C) at the second binding site.

A second sialic acid binding site at the NA domain dimer interface (Figures 5C, 8A) was revealed in the X-ray structure of NDV Kansas HN in complex with thiosialoside [24], [29]. This second site is not conserved across the paramyxovirus HN proteins, as shown by its absence in the atomic structure of PIV5 HN [22]. For hPIV3 an N-linked glycan masks a second receptor-binding site [30], [31]. However, for NDV, functional studies indicate that this second site does play a role in NDV attachment and entry [24], [29], [32], [33].

In the Ulster HN structure, we observe that residues E584-K587 in the C-terminal extension directly engage the second sialic acid binding site, indicating that this would be sterically blocked in HN_0_ (Figures 5C, 8A). Within the second site, interactions with sialic acid come from polar contacts with mainchain atoms, and through a conserved hydrophobic pocket, including residues G169, L552, F553 from one subunit, and F156, V517, and L561 from the second subunit (Figure 8B). In the thialoside complex, sialic acid forms hydrogen bonds with S519, R516, F156 and G169. In the Ulster HN structure, the mainchain oxygen of K587 forms a hydrogen bond with S519 and W586 interacts with R516 through hydrophobic contacts, which would block thialoside interactions (Figure 8C). In addition, the side chain of K587 occupies the hydrophobic pocket, whose residues are conserved in Ulster and Kansas HN proteins. The second receptor binding site would thereby be completely blocked by the HN_0_ C-terminus.

## Discussion

In this report, we demonstrate that the longer HN precursor, HN_0_, found exclusively in avirulent NDV strains such as NDV Ulster [19], [20], [21], is folded to an auto-inhibited state. The C-terminal extensions of the Ulster HN block the neuraminidase active sites and second sialic acid binding sites, both of which engage receptors on target cells, explaining the reduced hemadsorption and catalytic activities of the HN_0_ precursor. We further demonstrate that this auto-inhibition is dependent upon a disulfide bridge formed by C596 in the extension and that C596 is centrally located at an extensive interdomain interaction between the C-termini, which rises like a turret above the HN NA domain dimer. Both proteolysis and mutation of C596 lead to HN activation, indicating that disruption of the C-terminal extension structure releases HN from the auto-inhibited state.

Other NDV strains, such as D26 and Queensland [19], [20], [21], also express a 616 amino acid long HN precursor protein. Sequence features important to the structure of the extension observed here in Ulster HN are also conserved in these related HN proteins, indicating that they fold similarly. Of 45 amino acids in the C-terminal extension structure, 12 appear absolutely conserved, including C-terminal residues that engage the active site (S614, W615), residues that interact with the second site (G585, W586 and D588) and residues that form the turret structure and C-helix (V591, P593, F595, N600, T602, R605, E609). Other key residues are highly conserved, such as Y611, although C596 is not absolutely conserved and is replaced by arginine or serine in a subset (<10%) of available database sequences. Compensatory changes may stabilize the HN turret structure in the absence of C596, as this appears critically important for placing inhibitory C-terminal residues into the HN active site and N-terminal residues in the second sialic acid binding site. The observation that the C-terminal extension engages both of these receptor-binding sites further indicates that both sites are functionally important in vivo, as other studies have suggested [24], [29], [32], [33].

Although the longer HN_0_ is only found in avirulent NDV strains, one study with recombinant engineered virus suggested that expression of HN_0_ does not reduce virulence, as followed by intracerebral inoculation of 1 day old chickens [18]. However this study did not address the natural route of infection or spread to various internal tissues. NDV virulence has been repeatedly linked to the efficiency of F protein cleavage, which is associated with the presence of multiple basic (Arg/Lys) residues at its cleavage/activation site [16], [18], [34], [35]. Despite these observations, a subset of naturally occurring NDV strains have evolved to express the precursor HN_0_ and HN_0_ clearly has inhibited receptor binding and neuraminidase activities, which would be expected to influence infectivity, viral spread and virulence [14], [15], [19], [36]. Precursor HN_0_ proteins likely confer some overall selective advantage in a natural infection setting, perhaps reducing virulence or pathogenicity in the avian host in a manner that may beneficially promote virus survival in a population. The evolution of an auto-inhibited attachment protein, requiring proteolytic activation, appears to be unique to NDV within the larger paramyxovirus family, although other paramyxovirus attachment glycoproteins exhibit length variations which might have functional consequences. Given the dramatic range of virulence of NDV strains, which encompass symptoms from mild respiratory infections to 100% mortality, there may have been strong selective pressure to moderate NDV pathogenicity, resulting in this unique mechanism for the inhibition of HN functions that are essential to the virus life cycle.

## Materials and Methods

### Cells and plasmids

293T cells were maintained in Dulbecco's modified Eagle's medium (DMEM) supplemented with 10% fetal bovine serum (FBS). HeLa CD4-LTR-βgal cells were maintained in DMEM supplemented with 10% FBS, 200 µg/ml G418 (InvivoGen, San Diego, CA), and 100 µg/ml hygromycin B (InvivoGen).

The growth of NDV Ulster and whole cell RNA eztraction was performed by Drs. Daniel J. King and Claudio Afonso at the USDA Poultry Rserach Laboratory, Athens, Georgia. DF-1 cells were infected with NDV Ulster at a multiplicity of infection of 5 plaque forming untits/cell and total cell RNA extracted. The cDNAs encoding NDV Ulster F and HN were synthesized by reverse transcription-PCR using the whole cell RNA. The cDNAs were cloned into the eukaryotic expression vector pCAGGS [37] and their nucleotide sequence obtained using an Applied Biosystems 3100-Avant automated DNA sequencer (Life Technologies Corp., Carlsbad, CA). DNA encoding the Ulster HN ectodomain (residues 49–616) and head (NA) domain (residues 124–616) were cloned into the vector pHLsec [38]. HN sequences were preceded by an N-terminal 6 His tag and a thrombin cleavage site. Mutation of Ulster HN cysteine 596 to serine was carried out by 4 primer PCR.

### Transfection, metabolic radiolabeling and antibodies

HeLa CD4-LTR-βgal cells were transfected with pCAGGS Ulster HN or F using Lipofectamine Plus (Life Technologies Corp.) according to the manufacturer's protocol. 24 h post-transfection (p.t.) the cells were washed with DMEM without methionine and cysteine (DMEM met^−^ cys^−^), incubated in DMEM met ^−^cys^−^ for 30 min and labeled for 30 min with 100 µCi/ml EXPRE^35^S^35^S Protein Labeling Mix (PerkinElmer, Waltham, MA). Following the labeling period the cells were incubated for 2 h in DMEM supplemented with 2 mM methionine, 2 mM cysteine. For half the samples the medium was removed after 90 min and replaced with medium containing 5 µg/ml TPCK-treated trypsin (Worthington Biochemical Corp, Lakewood, NJ). Cell lysates were prepared and immunoprecipitation was carried out as described previously [39] using a rabbit polyclonal antibody (R9722) raised against NDV Australia Victoria HN ectodomain and a rabbit polyclonal antibody raised against residues 409–423 of Australia-Victoria F protein (R1680). The samples were analyzed by SDS-PAGE and the radioactivity visualized using a Fuji FLA-5100 imaging system (Fuji Medical Systems, Stamford, CT).

### Protein expression and purification

The NDV Ulster “head” and full ectodomain constructs were expressed by transient expression in 293T cells. Briefly, 293T cells in 10 cm dishes were transfected using 23 µg pHLsec HN head or pHLsec ectodomain and 34 µg 25 kDa branched polyethyleneimine (Sigma-Aldrich; St. Louis, MO) per plate, the supernatant media was collected at 3 days p.t., clarified by centrifugation (650×g) in an Allegra 6 centrifuge (Beckman Coulter) and filtered through a 0.2 µm filter. The pH was adjusted to pH 8.0 and the proteins were purified by affinity chromatography using nickel-nitrilotriacetic acid (Ni-NTA) agarose (Qiagen; Valencia, CA). The Ni-NTA columns were washed with buffer containing 10 mM and 50 mM imidazole and the proteins were eluted using buffer containing 250 mM imidazole. The proteins were further purified by size exclusion chromatography, using a Superdex S200 column, with a running buffer of 50 mM Tris, pH 8.0, 200 mM NaCl. The protein buffer was subsequently exchanged to 25 mM Tris, pH 7.5, 50 mM NaCl. The eluted proteins were >90% pure by SDS-PAGE analysis and Coomassie brilliant blue or silver staining.

### Sucrose gradient analysis

Ulster HN head NA domain or HN C596S NA mutant protein were layered on top of a 5–25% (W/V) sucrose gradient formed over a 60% sucrose cushion. The gradients were centrifuged in a SW41 swinging bucket rotor at 38,000 rpm for 16.75 h at 20°C using an Optima L80 XP ultracentrifuge (Beckman Coulter). Sixteen 0.75 ml fractions were collected and analyzed by SDS-PAGE under non-reducing conditions.

### Hemadsorption

HeLa CD4-LTR-βgal cells were transfected with pCAGGS Ulster HN or empty vector using Lipofectamine Plus (Life Technologies Corp.). 18–20 h p.t. the media was removed, the cells were washed twice in phosphate buffered saline containing calcium and magnesium (PBS^+^) and incubated at 37°C for 20 min in PBS^+^ or PBS^+^ containing 5 µg/ml TPCK-treated trypsin (Worthington Biochemical Corp). The cells were then placed on ice, washed several times with PBS^+^ and incubated for 2 h at 4°C with 1 ml of a 1% suspension of chicken red blood cells (RBCs) in PBS^+^. The cells were thoroughly washed with PBS^+^ and photographed using an inverted phase-contrast microscope (Diaphot; Nikon, Melville,NY) connected to a digital camera (DCS 760; Kodak, Rochester, NY). For quantification of hemadsorption, after washing to remove unbound RBC's, 0.5 ml of water was added and the cells were incubated for 2 h at 4°C. The absorption of the clarified supernatant was read at 540 nm using a Beckman Coulter DU 730 Life Sciences UV/Vis spectrophotometer (Beckman Coulter, Brea, CA).

### Neuraminidase activity assay

10 µl 12.5% dimethyl sulfoxide was added to the wells of a 96 well plate followed by 10 µl HN protein diluted in enzyme buffer (162.5 mM 2-(n-morpholino)ethanesulfonic acid (MES) pH 6.5, 5 mM CaCl_2_). 30 µl substrate (54.2 mM MES, 5 mM CaCl_2_, 0.167 mM 4-methylumbelliferyl-N-acetyl-α-D-neuraminic acid) was added and the plate was incubated at 37°C for 15 min with shaking. 150 µl of stop solution (0.014 M NaOH in 83% ethanol) was added and the fluorescence of the cleaved substrate was measured at excitation and emission wavelengths of 360 and 440 nm respectively using a Spectramax M5 plate reader (Molecular Devices, Sunnyvale, CA).

### Electron microscopy

Solutions of NDV HN (Ulster) at a concentration of approximately 5 mg/ml were absorbed onto 300 mesh copper grids covered with a carbon film that had been freshly glow discharged. Grids were stained with a 1% aqueous solution of uranyl formate, freshly prepared and filtered immediately prior to use. Grids were observed in a JEOL 1230 electron microscope operated at 100 kV and images were acquired with a Gatan 831 CCD camera, at the Biological Imaging Facility, Northwestern University, Evanston, IL.

### Crystallization

The NDV HN (Ulster) neuraminidase “head” domain protein was concentrated to ∼8 mg/ml in 25 mM Tris, pH 7.5, 50 mM NaCl. The crystallization condition was identified by screening with the Wizard crystallization kits (Emerald BioSystems, WA) set up with a Phoenix robot. For further optimization of the initial hit condition, the protein was crystallized at room temperature by the hanging drop vapor diffusion method. The drops contain an equal ratio of protein and precipitant consisting of 17% PEG 8000, 200 mM MgCl_2_, 100 mM Tris, pH 8.5. Crystals appeared with a rod shape with dimension of ∼80×400 µm. The harvesting and freezing buffer contained 20% PEG 8000, 200 mM MgCl_2_, 100 mM Tris, pH 8.5, and 15% glycerol.

### Data collection, structure determination, and refinement

The diffraction dataset was collected at the Argonne Advanced Photon Source NE-CAT, station 24IDE and processed to 3.5 Å using HKL2000 [40]. The NDV HN Kansas [26] monomer structure (PDB ID: 1E8T) was used as the search model to determine the initial phases. Model building and structure refinement were performed with Coot [41], Refmac [42] in the CCP4 package [43], and Phenix [44]. The model after molecular replacement was first refined by rigid body refinement with Refmac [42], followed by two rounds of restrained refinement without and with NCS restraints, where residues 124–569 in Chains A, B, C and D were restrained by NCS. The C-terminus was built both by Buccaneer [43] and manual modeling. Further refinement was carried out using Phenix [44], with NCS restraints at the beginning and TLS refinement throughout to the final model. TLS groups were generated automatically in Phenix, with each chain (A, B, C, and D) divided into groups of residues as follows: 124∶192, 193∶229, 230∶300, 301∶459, 460∶555, 556∶557, and 578∶615. The data collection and final refinement statistics are collected in Table 3. Coordinates and structure factors have been deposited in the RCSB under PDB ID code 4FZH.

### Protein alignments

Protein alignments were performed using MultAlin [45].
